# Previous Mental Load and Incentives Influence Anticipatory Arousal as Indexed by the Baseline Pupil Diameter in a Speech-in-Noise Task

**DOI:** 10.1177/23312165231196520

**Published:** 2023-10-17

**Authors:** Defne Alfandari, Michael Richter, Dorothea Wendt, Lorenz Fiedler, Graham Naylor

**Affiliations:** 1 School of Medicine, Mental Health and Clinical Neurosciences, Hearing Sciences – Scottish Section, 6123 University of Nottingham, Glasgow, UK; 2 School of Psychology, 4589 Liverpool John Moores University, Liverpool, UK; 3 Eriksholm Research Centre, 263099 Oticon A/S, Snekkersten, Denmark; 4 Hearing Systems, Department of Health Technology, Technical University of Denmark, Lyngby, Denmark; 5 170718 NIHR Nottingham Biomedical Research Centre, Nottingham, UK

**Keywords:** listening effort, pupillometry, speech-in-noise, motivation, listening-related fatigue

## Abstract

Listening effort and fatigue are common experiences when conversing in noisy environments. Much research has investigated listening effort in relation to listening demand using the speech-in-noise paradigm. Recent conceptualizations of listening effort postulate that mental fatigue should result in decreased arousal and a reluctance to invest further effort, particularly when the effort is not worthwhile. The aim of the study was to investigate the influence of fatigue on listening effort, in interaction with listening demands and motivation. To induce fatigue 30 adults with normal hearing completed a 40-minute long speech-in-noise task (“load sequence”). Pre- and post-load sequence listening effort was probed in easy and hard listening demands (individually adjusted signal-to-noise ratios); with high and low motivation (manipulated with monetary incentives). Subjective effort, estimated performance, and tendency to quit listening were collected using rating scales. Baseline pupil diameter and mean pupil dilation were recorded as indices of anticipatory arousal and objective effort. Self-reported effort and mean pupil dilation were overall larger during hard SNR as compared to easy SNR. Baseline pupil diameter declined from pre- to post-load sequence, suggesting an overall decrease in arousal. Monetary incentives had no influence on the baseline pupil diameter for the easy SNR condition, but for the hard SNR condition larger incentives led to larger baseline pupil diameter. These results suggest that anticipatory arousal may be influenced by fatigue and motivation effects. Models of listening effort should account for the independent influence of motivation and previous load on anticipatory arousal and effort in distinct parameters.

## Introduction

Listening to speech in the presence of background noise is known to be effortful. For example, when having a conversation in a noisy environment, we may struggle to keep the required level of concentration to understand our conversation partner. We may even not be willing to invest the required effort to understand the other. This may be particularly the case if we have been conversing for a long time already and feeling fatigued. Conversely, if the conversation is important, for example one that could lead to an increase in our income, then we may be motivated to keep up listening carefully despite the fatigue. Much research has been conducted on how listening effort—defined as the deliberate allocation of mental resources to overcome obstacles in goal pursuit when carrying out a task that involves listening (Pichora-Fuller et al., 2016) scales with changes in background noise (McGarrigle et al., 2014; Ohlenforst et al., 2018). Although real-life conversations in noise involve experiences of listening-related fatigue and motivation to listen, the influences of fatigue and motivation on listening effort have rarely been measured.

Mental fatigue is well known to arise after a period of sustained effort, presenting as a subjective struggle to further mobilize effort (Boksem & Tops, 2008; Pattyn et al., 2018). In a state of fatigue, habitual, automatic tasks can still be completed, but tasks that require more effort are observed to suffer (Persson et al., 2013; van der Linden, 2011; van der Linden et al., 2003). Importantly, in a state of fatigue, whether an individual continues to invest mental effort depends on their motivation (Boksem et al., 2006; Müller & Apps, 2019). Recent physiological research shows that in a state of fatigue effort mobilization may be higher when participants are offered incentives for performance (Gergelyfi et al., 2015; Herlambang et al., 2019; Hopstaken et al., 2015). To induce mental fatigue in the lab, tasks that tap into working memory resources, that are on average of 30 minutes duration, and that are individually adjusted to be in challenging difficulty are the most effective (Bafna & Hansen, 2021; O’Keeffe et al., 2020).

Recent models that explain listening effort and fatigue acknowledge the role played by motivation and fatigue on listening effort (Pichora-Fuller et al., 2016; Schneider et al., 2019). The Framework for Understanding Effortful Listening (FUEL; Pichora-Fuller et al., 2016) suggests that effort should depend on arousal (capacity). Mental fatigue is postulated to decrease arousal and motivation (Schneider et al., 2019). Low arousal states are postulated to reduce the capacity to exert effort (Kahneman, 1973; Pichora-Fuller et al., 2016). Based on theories of motivation, the FUEL also postulates that larger motivation should increase listening effort particularly when listening demands are high (Brehm & Self, 1989). Similarly, the quantitative model of listening-related fatigue (Schneider et al., 2019) postulates that in addition to listening demands (demanded effort), motivation (base motivation and actual motivation) should determine listening effort. Together, these models suggest that particularly in demanding listening situations, fatigue and motivation should influence arousal and listening effort.

To date listening effort has been measured using self-report, behavioral, and physiological methods (McGarrigle et al., 2014). Whereas self-report measures capture the subjective experience of effort, and thus are high on face validity, physiological measures assess objective effort and have larger sensitivity (McGarrigle et al., 2014). Thus, arguably, the use of multiple measures enables the capture of multiple dimensions of effort (McGarrigle et al., 2014).

One widely used physiological measure to assess listening effort is pupillometry (Naylor et al., 2018). The task-evoked pupil dilation has long been used to index mental effort (Beatty, 1982; Hess & Polt, 1964; Larsen & Waters, 2018; van der Wel & van Steenbergen, 2018). Within hearing sciences, pupillometry has commonly been combined with the speech-in-noise paradigm, where participants listen to sentences in noise and are asked to repeat the sentences (Winn et al., 2018). Whereas the baseline pupil diameter (BPD), which is commonly measured during the 1 second before the presentation of the sentences, is thought to index momentary arousal (Ayasse & Wingfield, 2020), the baseline-corrected task-evoked pupil dilation that follows the onset of the sentences is thought to index the effort invested in listening (Kramer et al., 1997; Winn et al., 2018).

Previous research shows that at the SNRs where sentence perception performance is around 80%, speech reception is subjectively reported as easy and elicits relatively small pupil dilation (Wendt et al., 2018). At the SNR where sentence perception accuracy is around 50% correct, speech reception is subjectively reported as challenging and elicits the largest pupil dilation as compared to the SNR where performance is poorer or better (Ohlenforst et al., 2017a; Wendt et al., 2018). Thus, the influence of fatigue and motivation on listening effort should be largest when listening in SNRs where performance is around 50%.

A few studies have investigated the influence of motivation on listening effort (e.g., Koelewijn et al., 2018, 2021; Richter, 2016; Zhang et al., 2019). Koelewijn offered normal hearing (NH) participants high (5 euros) or low (0.20 euro) rewards on the condition that they repeated 70% or more of the sentences correctly. This was done for blocks of 20 trials in quiet, and in babble noise at SNRs that were adapted from trial to trial, targeting 50% and 85% correct sentence recognition. Koelewijn et al. (2018) reported larger peak pupil dilation (PPD) with larger incentives for the conditions with babble noise, but not for the quiet condition. In a later investigation with the same babble noise and reward conditions, Koelewijn et al. (2021) reported no significant evidence for an effect of reward. However, they report post hoc analyses that suggest reward effects on the post-peak interval of the task-evoked dilation, only for the hard SNR condition. In both studies, participants reported larger effort during the 50%SNR condition, but the influence of incentives on self-reported effort did not reach significance. Although inconsistent, together these studies suggest that monetary incentives may modulate listening effort, particularly in lower SNRs.

One study has investigated the influence of daily-life fatigue on listening effort using pupillometry (Wang et al., 2018). In a sample consisting of both NH and HI adults, Wang et al. (2018) report a negative association between daily-life fatigue and the PPD. Due to the correlational nature of this finding, the mechanism driving this association remains unclear.

To date the influence of task-induced mental fatigue on listening effort has been investigated in adults in one study using pupillometry (McGarrigle et al., 2017b). McGarrigle et al. (2017b) used a sentence-to-picture verification task where NH participants indicated whether the content of a sentence that was presented in babble noise (65 dB) matched that of a picture presented to them subsequently. This task consisted of blocks of easy (+15 dB SNR) and hard (−8 dB SNR) trials. Using a growth curve analysis, the authors showed that particularly for the hard SNR condition, at the second 20 minutes half of the task, the decline of the pupil curve after the peak was steeper as compared to the first half. This finding, indicative of a reduction in arousal in the second half of the task, was attributed to mental fatigue. Self-reported effort was larger in the hard versus the easy listening, but there was no difference in self-reported fatigue with any of the conditions. These results suggest that during a speech-in-noise task, when assessed through pupillometry, mental fatigue should manifest as a reduction in arousal.

The main aim of the present study was to investigate the influence of task-induced mental fatigue on listening effort and arousal. To induce listening-related mental fatigue, normal hearing participants were asked to complete a 100-trial-long speech-in-noise test (“load sequence”) in individually adjusted challenging difficulty. Pre- to post-load sequence measures of arousal and effort were taken in hard and easy SNR (listening demand), and for High and Low incentive (motivation) conditions. Self-report measures of effort, estimated performance, and tendency to quit listening were collected using 10-point likert scales. In addition, BPD and baseline-corrected mean pupil dilation (MPD) were calculated as indices of arousal and listening effort. In light of the previous research summarized above, the following were hypothesized. Averaged across the motivation and SNR conditions, a pre- to post-load sequence decline in BPD (Hypothesis 1). Averaged across the motivation and probe time conditions, overall, larger ratings of effort and MPD in the hard SNR condition (Hypothesis 2). A decline in ratings of effort and MPD from pre- to post-load sequence that is largest for the hard SNR -low incentive condition (Hypothesis 3).

## Methods

### Participants

**Figure 1.**
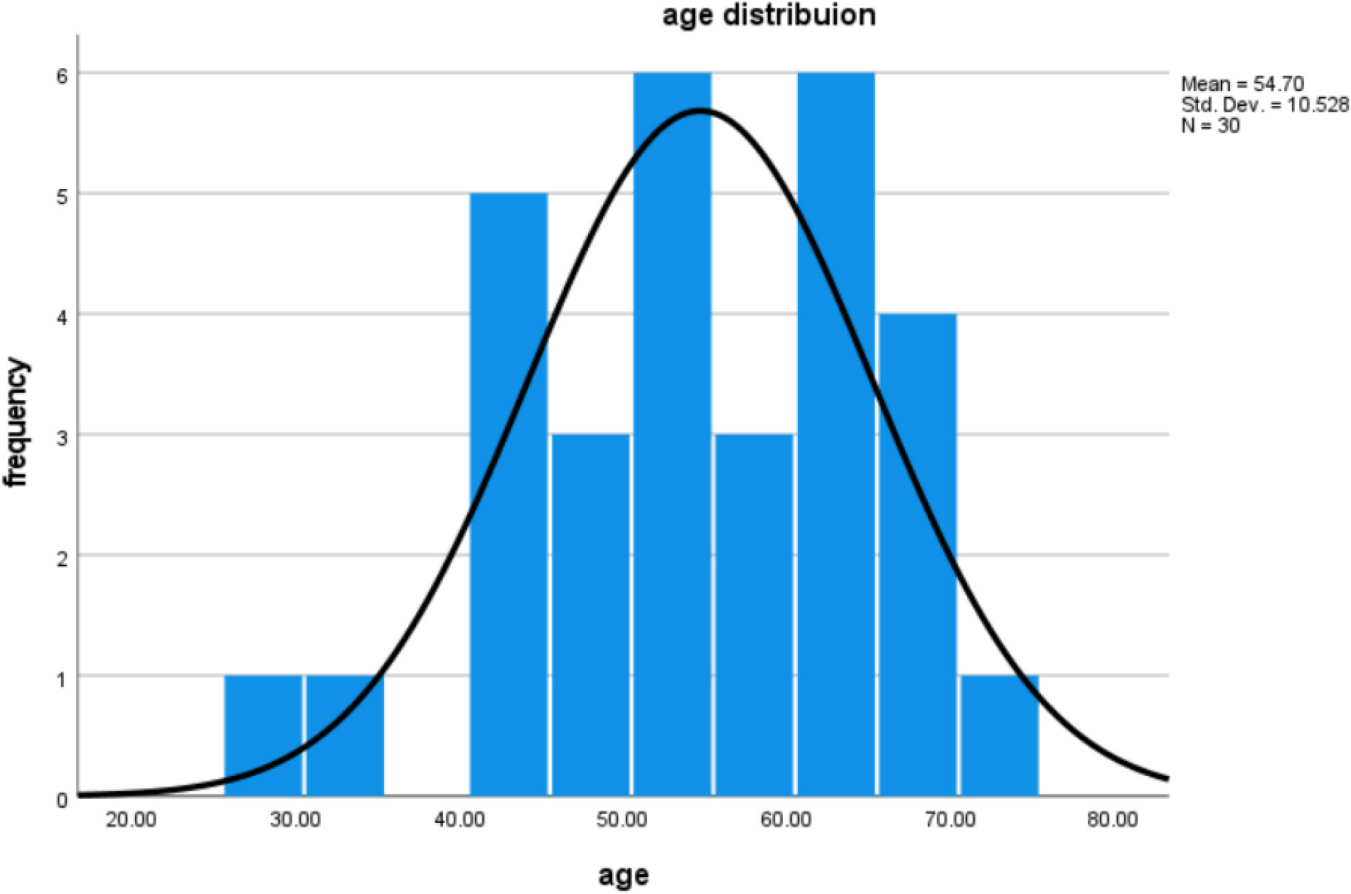
Age and frequency of age in the sample.

The data of 30 volunteers (age between 28 and 72 years, *M* = 54.67, *SD* = 10.91) are reported here. Figure 1 shows the age distribution of participants. A database search was done to select participants who fulfilled the inclusion criteria. Participants who had an audiogram with max 6 months old that indicated normal hearing were invited. All participants had normal hearing (i.e., hearing thresholds were less than or equal to 25 dB HL over the frequencies from 0.25 to 4 kHz for both ears). Participants reported normal or corrected-to normal vision and no neurological or psychiatric disorders. All participants were native speakers of English.

Participants were naïve to the fatigue-related aim of the experiment. This was to avoid fatigue-related interoceptive awareness interfering with the natural motivation to perform during the experiment. The study was approved by the ethics committee of the University of Nottingham, Faculty of Medicine and Health Sciences. All participants provided written informed consent. After completing their participation participants were debriefed about the true aims of the study.

We estimated the sample size based on a power calculation. To the best of our knowledge, this is the first study to experimentally investigate the fatigue × motivation × task-demand interaction effect in a speech-in-noise task. Based on a small-to-medium effect size of the motivation × listening demand × fatigue interaction (*d* = 0.35) on the pupil dilation and large power (1  - *β* = 0.95) we calculated that a sample size of 30 would be needed using the G*Power software (version 3.1.9.7). During our pilot testing, we noticed that the quality of our pupillometry recordings differed widely across participants. Thus, taking into account possible exclusions of participants based on data quality, an additional 16 participants were recruited. In total 46 participants were recruited from the database of the Hearing Sciences – Scottish Section, School of Medicine, University of Nottingham. Later, the data of 6 participants were excluded due to errors in the presented SNRs, and the data of 10 participants were excluded due to excessive missing samples (see a detailed description of data exclusion criteria below in section “pre-processing”).

#### Experimental Design

**Figure 2.**
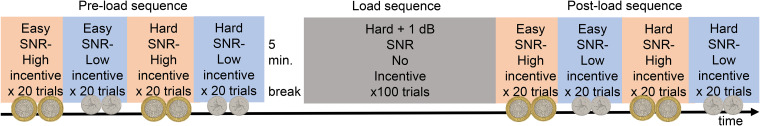
Experimental design. In a within participant design, adults with normal hearing completed a speech-in-noise task of 100 trials (“load sequence”). Pre- and post-load sequence listening effort was measured in blocks of 20 trials in easy and hard SNR (predetermined and fixed SRT80 and SRT50, respectively) conditions and with low and hard monetary incentives (0.4 and 4 GBP, respectively).

A pre-/post-fatigue experiment with a within-subject design was used to investigate the interactive effects of listening-related fatigue and motivation on listening effort. Figure 2 illustrates the experimental design. A challenging sustained speech reception task (i.e., “load sequence”) was used to induce fatigue. As previous literature indicates that tasks that tap into working memory, that are individually adjusted to be in challenging difficulty, and that are of 30 minutes long are successful in inducing fatigue, we used a speech-in-noise task of individually adjusted SNR, that was 30 minutes long to induce fatigue (Bafna & Hansen, 2021; O’Keeffe et al., 2020). We did not opt for a task that was longer than 40 minutes long due to ethical considerations. Listening effort was evaluated pre- and post-load sequence using pupillometry and self-report. Both pre- and post-load sequence, 4 blocks (i.e., 2 × motivation [high vs. low incentive] × listening demand [easy vs. hard SNR] of 20 trials each were administered. Per participant, the order of the pre-load sequence blocks was the same as the post-load sequence blocks. Across participants, the order of the conditions was randomized. After the last pre-load sequence block, participants took 5 minutes break to recover from the possibly fatiguing effects of the pre-load sequence blocks.

### Stimuli and Trial Sequence

The stimuli in the pre- and post-load sequence blocks consisted of everyday speech sentences from the Bamford–Kowal–Bench corpus (Bench et al., 1979) whereas those in the load sequence block consisted of sentences from the Institute of Hearing Research corpus (MacLeod & Summerfield, 1990). The sentences were read by a male speaker. The male speaker in the stimuli presented during the load task and the pre- and post-load tasks was the same speaker. The 4-talker babble masker noise consisted of speech segments from 2 female and 2 male talkers. The long-term average frequency spectrum of the masker noise was identical to that of the target speech signal.

**Figure 3.**
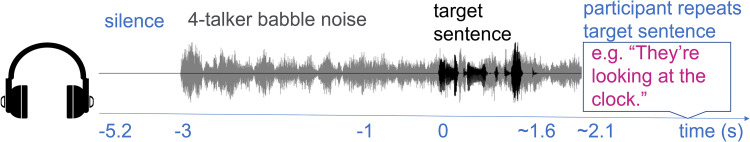
Trial sequence. A 4-talker babble noise started after the experimenter pressed the start button. After 3 seconds of noise, the target sentence was played. The target sentence was followed by 0.5 seconds of babble noise. Thereafter participants were asked to repeat the target sentence.

Figure 3 illustrates the trial sequence. Trials started 2.2 seconds after the experimenter pressed a button. A 4-talker babble noise (at 70 dB SPL for all trials) was presented. The target sentences (length: *M* = 1.62 seconds, *SD* = 0.40 seconds) started 3 seconds after the start of the babble noise. Due to a technical error that prevented the babble noise from continuing for longer, the babble noise continued for 0.5 seconds after the ending of the target sentences. Thereafter participants were prompted to repeat the sentences that they heard. The experimenter scored the sentence as correct when all the keywords were repeated correctly (sentence-based scoring) by a button-press. The button-press of the experimenter started the next trial.

### Subjective Report Scales to Assess Subjective Effort, Performance, and Tendency to Quit Listening

After each of the of 20-sentence blocks during pre- and post-load sequence, participants were asked to report how much effort they mobilized for listening, how well they thought they performed, and how much they felt an inclination for quitting listening (cf. Koelewijn et al., 2018). Three visual 10-point scales were printed on an A4 paper with the words “How much effort did understanding the sentences in the last block require?” (0 = no effort, 10 = very much effort), “How would you estimate the amount of sentences that you repeated correctly” (0 = none of the sentences, 10 = all of the sentences), “How would you rate your tendency for quitting listening because the sentence was too difficult?” (0 = This happened for none of the sentences, 10 = this happened for all of the sentences). To prevent participants from switching between the eye-tracker glasses to their reading glasses, which would be a break and a potential recovery from fatigue, the experimenter read the instructions aloud to the participants. Verbal responses from the participants were noted by the experimenter. Answers in fractional numbers (e.g., 3.5) were allowed.

### Apparatus

The sentences were presented through a software running on MATLAB (Matlab, 2016a) using the SoundMexPro tool. The audio output from the software was amplified using RME Babyface Pro audio interface and presented to the participants through circumaural AKG K-702 Harman High End Reference headphones. The speech reception test software was integrated with the Tobii Pro 2 Eye Tracker Glasses. The glasses were set to a sampling frequency of 100 Hz for the first 15 participants. Intermediate data quality checks revealed that a lower sampling rate led to more reliable data, such that the sampling frequency was later set to 50 Hz for the remaining participants. The pupil diameter of both eyes was recorded. Sessions took place in a sound-attenuated booth. The experimenter monitored the experimental stimuli, gaze position, and pupil recordings through two screens in an adjacent room. The experimenter listened to and scored the responses of the participants through a graphical interface created in MATLAB.

### Procedure

Prior to the experiment, participants received information about the study. The information focused on listening effort and motivation as the main research questions. Participants were verbally told that the experiment has no known side effects apart from mild fatigue. Participants visited our facilities twice within the course of 3 weeks. The first visit included air-conduction audiometry and ear examination, the estimation of SRTs to be used in the experiment, and a pilot eye-tracker calibration as a preparation for the second visit. The pre-/post-fatigue pupil recording was scheduled at the second visit. The first visit took approximately 40 minutes whereas the second visit was 2 to 2.5 hours long.

The SNRs corresponding to SRT50 and SRT80 were estimated in two adaptive speech-in-noise procedures. The adaptive procedure for both SRT50 and SRT80 was run twice. The first run was recorded as practice whereas the resulting SNRs from the second run were noted to be used in the experiment in the second visit. During each adaptive track 30 sentences from the BKB corpus were presented (Bench et al., 1979). Throughout the whole procedure the intensity of the background masker was 70 dB (as averaged across 30 seconds). During the estimation of SRT50 the first sentence was presented at 70 dB and increased by 4 dB until the participants repeated all keywords correctly (sentence scoring). The intensity of the following three sentences were decreased or increased by 4 dB, depending on whether the participants repeated all the keywords in the sentence correctly or not, respectively. The intensity of the fifth sentence was calculated by taking the average of the intensity levels of the first four sentences and the intensity level of what the fifth sentence would have been with a 4 dB stepsize. For the rest of the sentences, the level of the target sentence was decreased or increased by 2 dB depending on correct or incorrect (incomplete) repetition of the keywords, respectively. The estimated SRT50 was calculated as the average SNR of the last five sentences and what an extra sentence would have been presented at. The SRT80 was estimated with a similar procedure to that of SRT50, where the stepsizes for the first four sentences were −1.6 dB in correct and +6.4 dB in incorrect recognition. After the 5^th^ sentence, the stepsizes were −0.8 and +3.2 dB, respectively.

The second visit started with the eye-tracker calibration. Participants wore the Tobii Pro glasses and looked at a fixation cross card that was placed approximately 1 meter away from them. They were asked to keep their gaze on the card throughout the experiment and to schedule their blinks after the end of the target sentences. The illumination in the booth (LED strip lighting) was adjusted for the dynamic range of the pupils of each participant (cf. Zekveld et al., 2010; (cf. Winn et al., 2018)). For this, pupil size was recorded for 30 seconds in dark and 30 seconds in maximum illumination (750 lux as measured when the light meter faced the wall). The light was adjusted with a dimmer switch in 30-second long recordings until the elicited pupil size was approximately half-way between those in dark and maximum illumination. After this light adjustment, participants practiced the speech-in-noise task with 10 sentences in the easy SNR and 10 sentences in the hard SNR. The aim of the practice round was firstly to remind participants of the speech-in-noise procedure and, secondly, to give the participants an understanding of the task demands in the easy and hard SNR conditions. To this end, during the practice round participants received feedback on how many sentences they repeated correctly. Sentences were scored as correct when all the keywords had been repeated correctly.

Participants were told that they could earn additional monetary reward by correctly repeating minimum 14 of the sentences out of blocks of 20 sentences. Before each of the 20-trial blocks, written reminders about the task-demand and monetary reward (e.g., “-Difficult- -£4-” in font-size 35) were placed at the visual periphery of the participants. Participants were informed beforehand that this session would take approximately 2 hours, but were not informed about how many blocks of trials the experiment had in total. After completing the pre-load sequence blocks, participants took a 5-minute long break, where they could take the eye-tracker glasses off. The breaks were mostly spent conversing with the experimenter in quiet. At the beginning of the load sequence, participants were told that the coming block did not entail any rewards, and that this block would be slightly easier than the hard SNR blocks and somewhat longer than the previous blocks. Immediately after the load sequence, participants continued with the post-load sequence blocks. Throughout the experiment, participants could see the experimenter who sat outside of the booth in the periphery of their vision.

#### Pre-Processing and Calculation of the Pupil Dilation Indices

Pupil data were processed using MATLAB R2018b (MathWorks, Natick, MA). Pupil traces starting 1 second before the presentation of the target sentence until the end of the target sentence were selected for analyses. All traces were re-sampled to 50 Hz to eliminate regular artifacts that were observed in some of the traces by visual inspection. The aim of down sampling was to increase the quality of the data as suggested by technical experts. The 100 Hz was achieved through sampling through two different cameras in alternation. We believed that data quality and accuracy would increase if data was sampled through a single camera. Traces with more than 30% of missing data were considered invalid and excluded from the analyses (cf. Wendt et al., 2018). The data of a given participant was included in the analyses if 10 or more of the trials per block of 20 trials of each experimental condition were valid. For the excluded participants, the number of excluded blocks was not larger in the post-load block as compared to the pre-load block.

Pupil diameter values more than 2 standard deviations away from the median, and consecutively missing samples in each trial were coded as blinks. Blinks were then interpolated in a linear fashion. The interpolation started for the samples within 35 ms before and ended for the samples within 100 ms after the blink to account for blink-related changes in pupil size. Later a moving average filter with a window of 15 samples (300 ms) was used to smooth the de-blinked trials and to remove any high-frequency artifacts (Winn et al., 2018).

For each trial, the mean value of the pupil trace corresponding to the 1-second period before the onset of the target sentence was taken as baseline pupil dilation (BPD; Winn et al., 2018) The average pupil size during the presentation of the target sentence minus the baseline pupil size for each trial was taken as the MPD. Here we chose to focus on MPD, and not the PPD. Whereas these metrics are not the same, they are comparable (Winn et al., 2018). The PPD requires a longer retention interval (>3 seconds) between the end of stimulus presentation and beginning of the repetition of the stimuli by the participant. Our trials included shorter retention intervals (0.5 seconds; see also procedure section above) to ensure a faster pace and prevent any recovery from fatigue. Therefore MPD was favored.

### Statistical Analyses

The dependent measures of interest (performance accuracy, self-rated effort, performance, quitting tendency, BPD, and MPD) were each entered in a 3-way repeated-measures analysis of variance (ANOVA) in SPSS with probe time (pre- vs. post-load sequence), SNR (easy vs. hard), and monetary incentives (high vs. low) as independent variables.

## Results

### Speech Reception Thresholds

**Figure 4.**
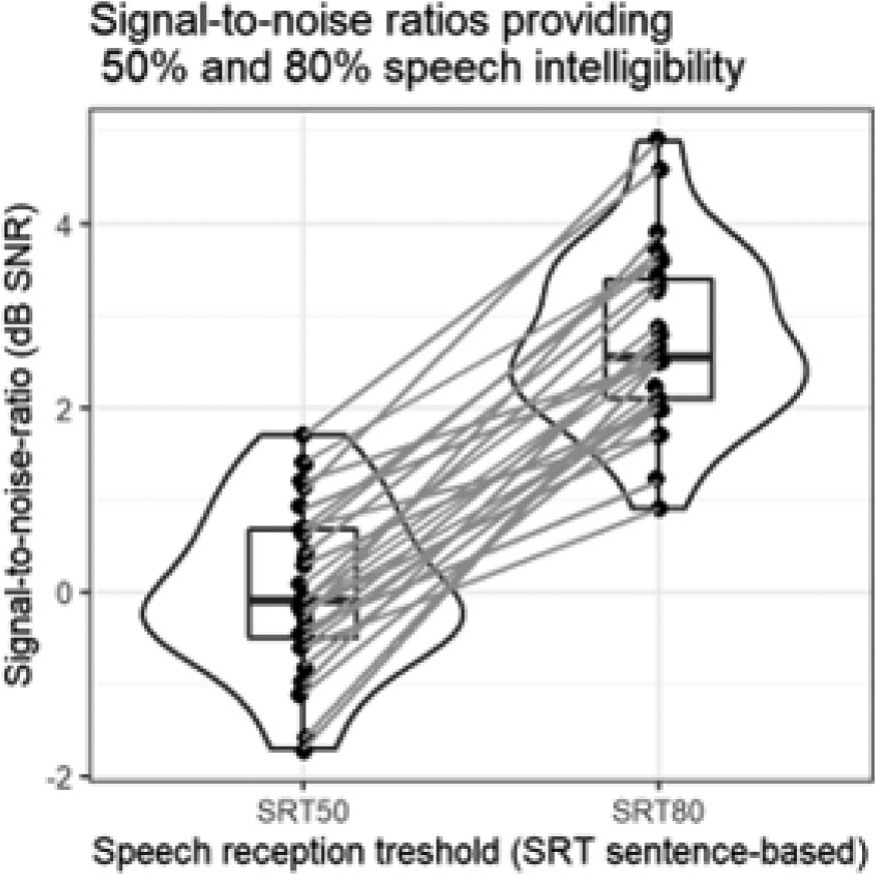
SRT50 and SRT80 of the participants. These were later used in the hard and easy conditions of the experiment, respectively.

Figure 4 shows the estimated SRT50 and SRT80 values in dB SNR for each participant. These were later used in the hard SNR and easy SNR conditions of the experiment, respectively. The estimated SRT50 (*M* = 0.03, *SD *= 0.87) was statistically significantly lower than that of SRT80 (*M* = 2.76, *SD* = 0.93) [*t*_paired_ (29) = 18.210, *p *< .001]. The difference between SRT80 and SRT50 was larger than zero for all of the participants (*M* = 2.73, *SD *= 0.87).

### Sentence Recognition Performance

Table 1 shows sentence recognition performance as a function of probe time (pre- vs. post-load sequence), monetary incentive (High vs. Low), and SNR (easy vs. hard). The repeated-measures ANOVA showed a main effect of SNR on speech recognition performance [*F*(1, 29) = 101.706; *p *< .001; $\eta_{p}^{2}=0.778$ ]; speech recognition performance was better in the easy SNR condition. There was no significant evidence for effects of probe time [*F*(1, 29) = 0.513; *p *= .480; $\eta_{p}^{2}=0.017$ ] or monetary incentive [*F*(1, 29) = 1.629; *p *= .212; $\eta_{p}^{2}=0.053$ ]. All terms for interaction effects on performance were non-significant: probe time × monetary incentive interaction effect [*F*(1, 29) = 0.003; *p *= .956; $\eta_{p}^{2}<0.001$ ]; the SNR × probe time interaction [*F*(1, 29) = 0.719; *p *= .403; $\eta_{p}^{2}=0.024$ ]; SNR × monetary incentive [*F*(1, 29) = 0.271; *p *= .607; $\eta_{p}^{2}=0.009$ ]; probe time × SNR × monetary incentive [*F*(1, 29) = 0.115; *p *= .737; $\eta_{p}^{2}=0.004$ ]. In sum, there was only significant evidence for the effect of SNR on performance.

**Table 1.** Sentence Recognition Performance.

Probe time	Pre-load sequence		Post-load sequence	
Incentives	High	Low	High	Low
Easy SNR	87 (9)	89 (2)	88 (10)	88 (15)
Hard SNR	66 (16)	67 (13)	67 (11)	70 (13)

Mean speech-in-noise performance (%) for the SNR, probe time, and monetary incentive conditions (SD in parentheses). Sentences were scored as correct when all keywords had been correctly repeated.

### Self-Rated Effort

**Figure 5.**
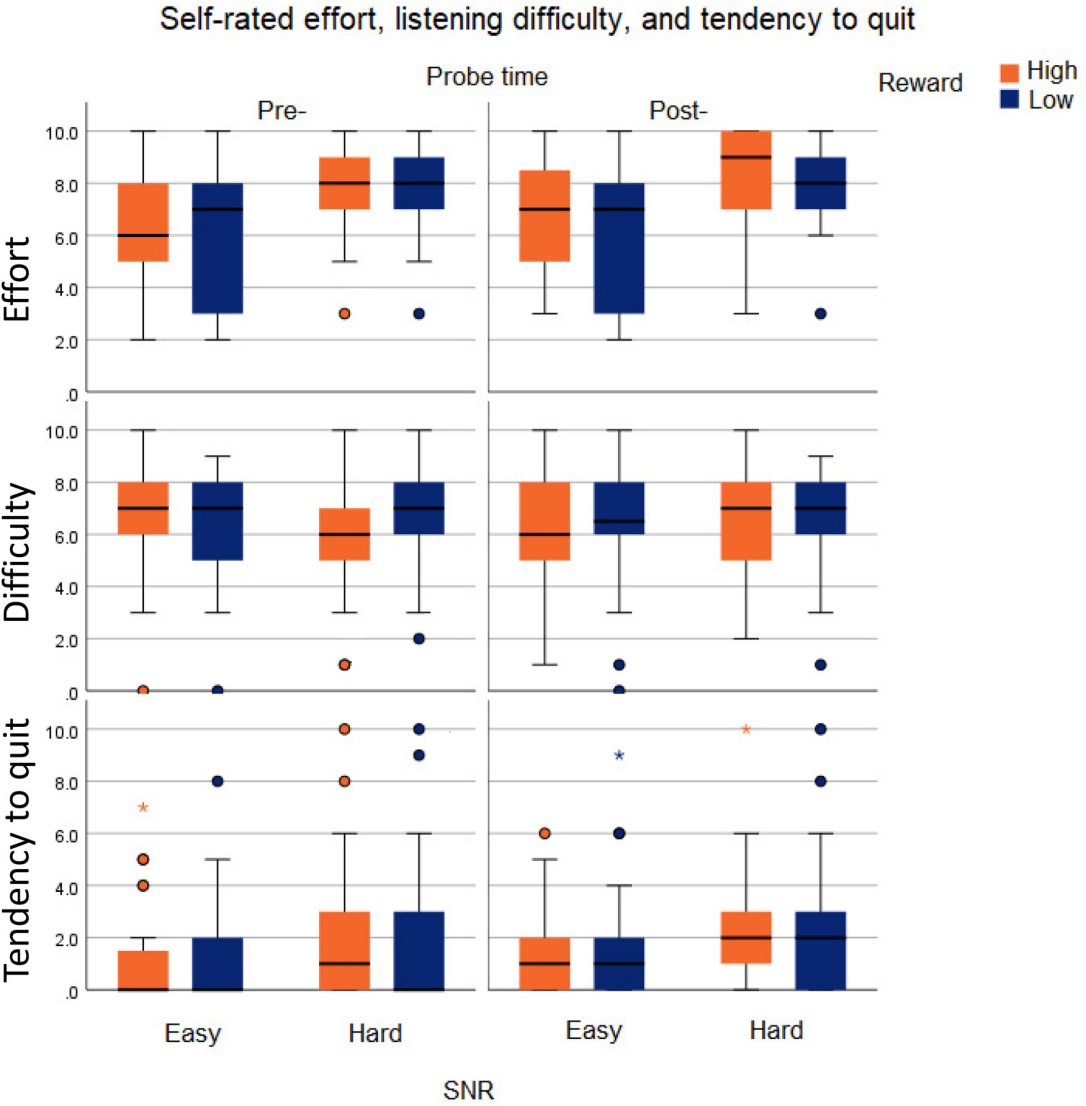
Boxplots of self-rated effort, performance, and tendency for giving up listening for the SNR, monetary incentive, and probe time conditions. Shapiro–Wilk tests of normality confirmed that all the rating scores were normally distributed (all *p*'s < .05).

The repeated measures ANOVA with self-rated effort as the dependent variable showed (See Figure 5) a main effect of SNR [*F*(1, 28) = 37.384; *p *< .001; $\eta_{p}^{2}=0.572$ ], as participants reported putting more effort in the hard SNR condition. There was a significant main effect of monetary incentive on self-rated effort [*F*(1, 29) = 1.793; *p *= .191; $\eta_{p}^{2}=0.060$ ] as participants reported larger effort after the high monetary incentive blocks. Although on average participants appeared to rate greater effort in the post-load sequence blocks as compared to the pre-load sequence blocks, the effect of probe time on self-rated effort did not reach significance [*F*(1, 29) = 1.793; *p *= .191; $\eta_{p}^{2}=0.060$ ]. The effect of SNR on self-rated effort did not depend on monetary incentive [*F*(1, 29) = 0.155; *p *= .697; $\eta_{p}^{2}=0.005$ ], or on probe time [*F*(1, 29) = 0.050; *p *= .825; $\eta_{p}^{2}=0.002$ ]. The effect of monetary incentive on effort did not depend on probe time [*F*(1, 29) = 0.602; *p *= .444; $\eta_{p}^{2}=0.021$ ]. Nor was there a significant SNR × monetary incentive × probe time interaction [*F*(1, 29) = 1.077; *p *= .308; $\eta_{p}^{2}=0.037$ ].

### Self-Rated Performance

The repeated measures ANOVA with self-rated performance as the dependent variable showed (see Figure 5) a main effect of SNR, [*F*(1, 29) = 27.282; *p *< .001; $\eta_{p}^{2}=0.494$ ] as participants rated their performance as poorer in the hard SNR condition. The main effects of monetary incentive [*F*(1, 29) = 0.389; *p *= .538; $\eta_{p}^{2}=0.014$ ] and probe time [*F*(1, 29) = 0.055; *p *= .817; $\eta_{p}^{2}=0.002$ ] were not significant. The effect of SNR did not depend on monetary incentive [*F*(1, 29) = 0.014; *p *= .907; $\eta_{p}^{2}<0.001$ ] or probe time [*F*(1, 29) = 0.541; *p *= .468; $\eta_{p}^{2}=0.019$ ]. The monetary incentive × probe time interaction effect was not significant [*F*(1, 29) = 0.090; *p *= 0.766; $\eta_{p}^{2}=0.003$ ]. Nor was the SNR × probe time × monetary incentive interaction significant [*F*(1, 29) = 2.598; *p *= .118; $\eta_{p}^{2}=0.085$ ].

### Self-Rated Tendency to Quit

The repeated measures ANOVA with self-rated tendency for quitting as the dependent variable (see Figure 5) showed a main effect of SNR [*F*(1, 29) = 12.689; *p *< .001; $\eta_{p}^{2}=0.312$ ], as on average, self-rated quitting was greater in the hard SNR condition. There was no significant main effect of monetary incentive [*F*(1, 29) = 0.646; *p *= .428; $\eta_{p}^{2}=0.023$ ]. Although the average tendency for quitting appeared to be larger at all post-load sequence blocks as compared to the corresponding pre-load sequence blocks, the main effect of probe time did not reach significance [*F*(1, 29) = 2.358; *p *= .136; $\eta_{p}^{2}=0.0789$ ]. The effect of SNR on tendency to quit did not depend on monetary incentive [*F*(1, 29) = 0.005; *p *= .945; $\eta_{p}^{2}<0.001$ ] or on probe time [*F*(1, 29) = 0.289; *p *= .595; $\eta_{p}^{2}=0.010$ ]. There was no monetary incentive × probe time interaction [*F*(1, 29) = 0.028; *p *= .869; $\eta_{p}^{2}=0.001$ ]. The average increase in self-reported tendency for quitting from pre- to post-load sequence appeared largest in the hard SNR – low monetary incentive block, but the 3-way SNR × monetary incentive × probe time interaction did not reach significance [*F*(1, 29) = 2.048; *p *= .163; $\eta_{p}^{2}=0.068$ ].

### Baseline Pupil Diameter

**Figure 6.**
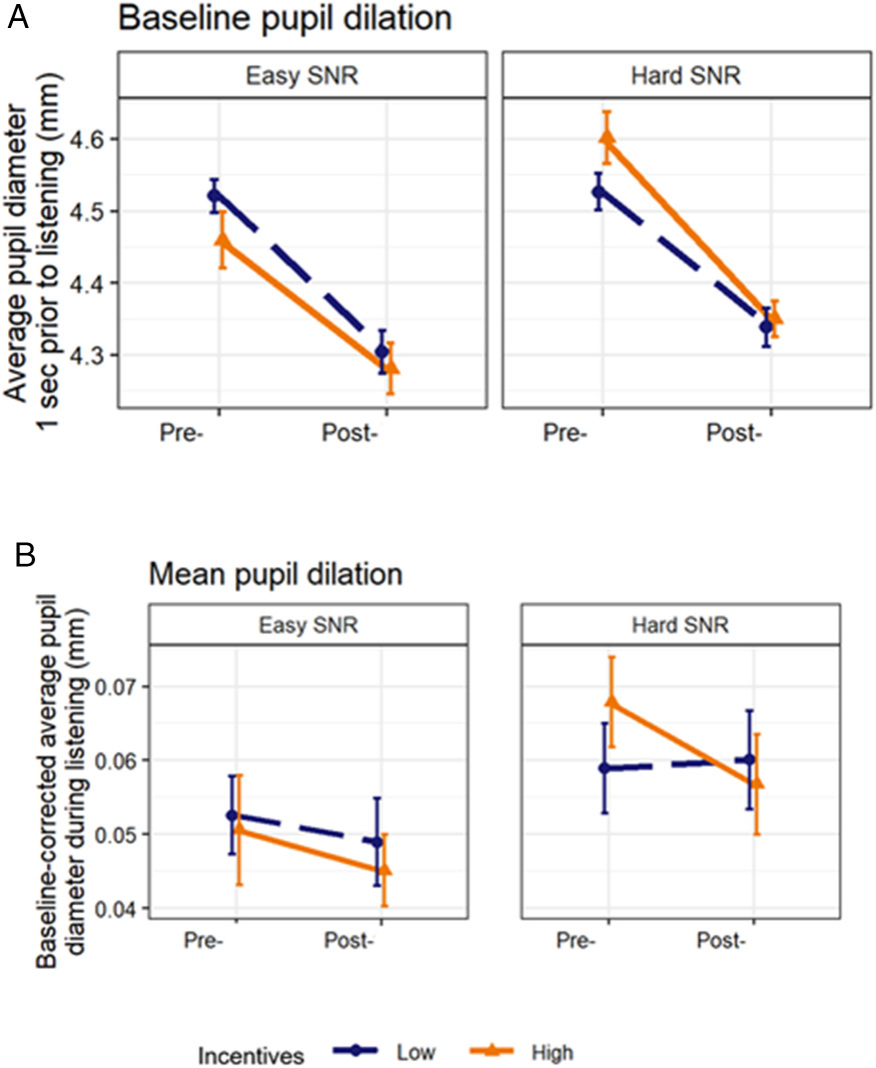
Baseline pupil diameter and baseline-corrected mean pupil diameter during listening to the target sentences for the SNR, probe time, and monetary incentive conditions.

Figure 6A shows BPD as a function of SNR (easy vs. hard), monetary incentive (High vs. Low), and probe time (pre- vs. post-). A repeated-measures ANOVA showed a main effect of SNR, as BPD was larger in anticipation of hard trials as compared to that in anticipation of easy trials, [*F*(1, 29) = 13.790; *p* < .01; $\eta_{p}^{2}=0.332$ ]. While there was no main effect of monetary incentive on the BPD [*F*(1, 29) = 0.002; *p* = .964; $\eta_{p}^{2}<0.001$ ], there was a main effect of probe time on BPD, as BPD was larger in the pre-load sequence condition [*F*(1, 29) = 25.573; *p* < .001; $\eta_{p}^{2}=0.469$ ]. The effect of probe time on BPD did not depend on monetary incentive [*F*(1, 29) = 0.281; *p* = .600; $\eta_{p}^{2}=0.010$ , or on SNR [*F*(1, 29) = 0.645; *p* = .429; $\eta_{p}^{2}=0.022$ ]. We observed a monetary incentive × SNR interaction effect [*F*(1, 29) = 5.312; *p* < .05; $\eta_{p}^{2}=0.155$ ]. Post hoc analyses showed that there was no evidence for an effect of SNR on BPD in the low monetary incentive condition [*F*(1, 29) = 0.865; *p* = .360; $\eta_{p}^{2}=0.029$ ], but in the high monetary incentive condition, BPD was larger in the hard SNR [*F*(1, 29) = 14.015; *p* < .05; $\eta_{p}^{2}=0.326$ ] as compared to the easy SNR. The three-way SNR × probe time × monetary incentive interaction effect on BPD did not reach significance [*F*(1, 29) = 1.807; *p* = .189; $\eta_{p}^{2}=0.059$ ].

### Mean Pupil Dilation

Figure 6B shows MPD for the SNR, probe time, and monetary incentive conditions. A 2 × 2 × 2 repeated measures ANOVA showed a significant main effect of SNR on the MPD, as the MPD was larger in the hard SNR condition [*F*(1, 29) = 10.481; *p *< .01; $\eta_{p}^{2}=0.265$ ]. There was no main effect of monetary incentive [*F*(1, 29) < 0.001; *p *= .992; $\eta_{p}^{2}<0.001$ ], or probe time [*F*(1, 29) = 0.581; *p *= .452; $\eta_{p}^{2}<0.020$ ] on the MPD. The effect of SNR on the MPD did not depend on monetary incentive [*F*(1, 29) = 1.118; *p *= .299; $\eta_{p}^{2}<0.037$ ] or on probe time [*F*(1, 29) = 0.001; *p *= .970; $\eta_{p}^{2}<0.001$ ]. The monetary incentive × probe time interaction effect on the MPD was not significant [*F*(1, 29) = 1.118; *p *= .229; $\eta_{p}^{2}<0.037$ ]. Nor was there a significant 3-way SNR × monetary incentive × probe time interaction effect on MPD [*F*(1, 29) = 0.597; *p *= .446; $\eta_{p}^{2}<0.020$ ].

### Correlations Between Self-Report Measures and Pupil Indices

To explore the relationships between the subjective and objective measurements of effort, two repeated measures correlations were computed, where the MPD was the dependent variable. Self-rated effort, and self-rated tendency for quitting were independent variables. Participant numbers were added as random slope. The analyses showed a positive association between MPD and subjective effort scores [*F*(12, 222.885) = 2.019; *p* < .05], and a positive association between MPD and subjective tendency for quitting [*F*(12, 186.269) = 1.806; *p *< .05].

### Age Effects

Given the large age range of the participants, we sought to investigate whether the SNR × monetary incentive × probe time interaction on BPD and MPD depended on age, we added the variable age to the repeated measures analyses described above. For this, age was coded as a dummy variable, where participants were split into two groups (i.e., older and younger adults) depending on whether they were younger or older than the mean age of the sample. The analyses showed no statistically significant effect of age *D* [*F* (1, 28) = 3.232; *p* = .634; $\eta_{p}^{2}=0.008$ ], age × probe time interaction [*F* (1, 28) = 0.58; *p* = .812; $\eta_{p}^{2}=0.002$ ], age × SNR interaction [*F* (1, 28) = 0.176; *p* = .678; $\eta_{p}^{2}=0.006$ ], age × monetary incentive interaction [*F* (1, 28) = 0.19; *p* = .666; $\eta_{p}^{2}=0.007$ ], age × probe time × SNR interaction [*F* (1, 28) = 1.741; *p* = .198; $\eta_{p}^{2}=0.059$ ], age × probe time × monetary incentive interaction [*F* (1, 28) = .429; *p* = .518; $\eta_{p}^{2}=0.015$ ], age × SNR × monetary incentive interaction [*F* (1, 28) = 3.2; *p* = .084; $\eta_{p}^{2}=0.103$ ], or age × probe time × SNR × monetary incentive interaction [*F* (1, 28) = 0.484; *p* = .492; $\eta_{p}^{2}=0.017$ ] on BPD.

Similarly we observed no statistically significant effect of age *D* [*F* (1, 28) = 0.949; *p* = .338; $\eta_{p}^{2}=0.033$ ], age × probe time interaction [*F* (1, 28) = 0.51; *p* = .481; $\eta_{p}^{2}=0.481$ ], age × SNR interaction [*F* (1, 28) = 0.037; *p* = .849; $\eta_{p}^{2}=0.001$ ], age × monetary incentive interaction [*F* (1, 28) = 0.19; *p* = .666; $\eta_{p}^{2}=0.007$ ], age × probe time × SNR interaction [*F* (1, 28) = 2.837; *p* = .103; $\eta_{p}^{2}=0.092$ ], age × probe time × monetary incentive interaction [*F* (1, 28) = 2.405; *p* = .132; $\eta_{p}^{2}=0.079$ ], age × SNR × monetary incentive interaction [*F* (1, 28) = 1.699; *p* = .203; $\eta_{p}^{2}=0.103$ ], or age × probe time × SNR × monetary incentive interaction [*F* (1, 28) = 1.279; *p* = .268; $\eta_{p}^{2}=0.44$ ] on MPD.

## Discussion

The main aim of this study was to investigate the effect of task-induced fatigue on listening effort in interaction with listening demands and motivation. A sample of adults with normal hearing (NH) engaged in a challenging sustained speech-in-noise-task (SIN; “load sequence”). The load sequence was assumed to have induced mental fatigue. Pre- and post-load sequence listening effort was probed during speech-in-noise (SIN) tasks in 2 SNR (hard and easy) and 2 monetary incentive (Low and High) conditions. Self-report scales and pupil metrics were used to gauge arousal and listening effort. Averaged across the SNR and monetary incentive conditions, we expected a decline in arousal from pre- to post-load sequence (Hypothesis 1). Furthermore, we expected larger listening effort in the hard SNR condition as compared to the easy SNR condition (Hypothesis 2). Lastly, we expected listening effort to decline from pre- to post-load sequence, and more so for the hard SNR -Low monetary incentive condition (Hypothesis 3).

In line with the expected decline in arousal (Hypothesis 1), BPD was smaller post-load sequence as compared to pre-load sequence. Diminished alertness over time-on-task (Hopstaken et al., 2015) and reduced preparation for effortful trials (Ackerman, 2010; Boksem et al., 2006; Lorist, 2008; Lorist et al., 2000; McGarrigle et al., 2017a, 2017b) have typically been considered hallmarks of mental fatigue. This result is in line with previous reports of diminished arousal over a long listening task (McGarrigle et al., 2017b). It is also in line with the predictions of models that seek to explain listening effort and fatigue (Pichora-Fuller et al., 2016; Schneider et al., 2019).

Arousal has commonly been considered in relation to performance, where average levels of arousal elicit best performance (Unsworth & Robison, 2016). In the current experiment there was no significant evidence for a change in performance from pre- to post-load sequence. Although not significant, there was an increase in performance from pre- to post-load sequence in the hard SNR condition. Thus the decline in BPD could at least partially be attributed to habituation (Metalis & Hess, 1981; Verbaten et al., 1986) or learning effects. Given the difficulty in ruling out any of these effects, a blending of fatigue, habituation, and learning, which is common in previous investigations of mental fatigue (Faber et al., 2012; Hopstaken et al., 2015; van der Linden et al., 2003), may perhaps be another plausible interpretation for the overall decline in BPD.

No specific hypotheses were constructed regarding the influence of SNR and monetary incentives on the BPD, but given that the MPD is a baseline-corrected measure, the influences of SNR and monetary incentives on the BPD (i.e., our reference measure) were also investigated. Our analyses revealed that BPD was influenced by SNR, such that in the Low monetary incentive condition there was no effect of SNR on BPD, but in the High monetary incentive condition BPD was larger for the hard SNR condition. That is, BPD was larger in anticipation of difficult trials of high value. This suggests that motivation may affect the pro-active (pre-emptive) allocation of capacity in anticipation of listening (Braver, 2012; Jahfari et al., 2012). This result supports the view that the BPD in the speech-in-noise paradigm may not reflect a unitary construct (i.e., a one-to-one mapping between a psychological state and BPD does not seem plausible; Ayasse & Wingfield, 2020).

Supporting the hypothesis of larger listening effort in the hard SNR condition as compared to easy SNR (Hypothesis 2), we observed on average larger self-reported listening effort during the hard SNR condition, suggesting larger subjective effort. Furthermore, MPD was larger in the hard SNR condition as compared to the easy SNR condition. These result are in line with the previously reported effects of listening demand on listening effort (Ohlenforst et al., 2017b; Wendt et al., 2018) and with the predictions of models that seek to explain listening effort (Pichora-Fuller et al., 2016; Schneider et al., 2019). In addition to larger self-reported effort in the hard SNR condition, the self-reported tendency for quitting was also larger for the hard SNR condition. This supports the understanding that listening effort involves a deliberate component for overcoming obstacles as defined in the FUEL (Pichora-Fuller et al., 2016).

Contrary to the hypothesized mental fatigue × listening demand × motivation interaction (Hypothesis 3), there was no significant evidence for an SNR × probe time × monetary incentive interaction effect on self-reported effort. Nor was the 3-way interaction on the MPD significant. One factor to contribute to the lack of the interaction effect may be the incentives being insufficient to keep participants motivated in the post-load sequence condition. There was no main effect of monetary incentives on MPD either. The amount of monetary incentives was based on those previously reported by Koelewijn et al. (2018); however, the current participant sample was much older than those of Koelewijn et al. As older age is known to reduce sensitivity to monetary incentives (Dhingra et al., 2020), future studies with similar age groups could use larger or more intrinsic incentives. Previously Koelewijn reported influence of monetary incentives on PPD, but Koelewijn et al., (2021) reported lack of evidence for the influence of monetary incentives on PPD. Together these results suggest that the influence of monetary incentives on the pupil dilation response may not be a robust finding. In the current study, self-rated effort was significantly larger with higher incentives. Note that the wording of the effort scale tapped more into the demanded effort rather than the exerted one; thus one interpretation of this finding may be the thinking in listeners that they were asked by the experimenter to exert more effort in the high incentive condition. Also, to note is that average estimated performance (63%) was much lower than actual performance (78%). The level of performance required to obtain rewards (70%) may have been perceived as unachievable, diminishing post-load effort for regardless of the motivation condition.

Another factor to contribute to the insufficient evidence for the SNR × probe time × monetary incentive interaction effect on listening effort may be that the post-load sequence listening demands in the current study were not as demanding as planned. Although the SNR in the hard condition was predetermined and fixed at SRT50, average performance was 67% (sentence-based) correct. Thus, even after performing the load sequence, the hard SNR condition may have been achievable, thus eliciting listening effort. Although participants practiced with 30 trials before the adaptive SRT estimation track, this practice may have been insufficient, leading to the (non-significant) improvements that were observed. Future studies may opt for longer practice periods before running the adaptive estimation procedure. On the other hand, although not statistically significant, self-rated effort showed an increasing trend from pre- to post-fatigue for all the incentive and SNR conditions, suggesting that participants may have felt the need for compensatory effort in the post-load sequence block in order to keep up. Due to time constraints and ethical considerations the fatigue manipulation was not strong; participants may have been able to exert effort even after the manipulation. Furthermore, the short periods in between the trials may have allowed participants to rest between the trials (Nobre & Van Ede, 2018; Shalev & Nobre, 2021) and thereby mobilize enough capacity even after the fatigue manipulation.

Although the SNR × monetary incentive × probe time interaction on the self-reported tendency to quit did not reach statistical significance, the size of this effect was medium; on average participants rated largest tendency to quit in the post-fatigue-hard SNR-low Incentive block. This result suggests that in our sample of participants, on average, previous load sequence and monetary incentives may have contributed to the willingness to exert listening effort. In the current study participants may have been reluctant to express the tendency to quit as a result of politeness. Future studies may look into more indirect ways of gauging this tendency.

It is important to note that the current study included individually adjusted SNR levels throughout the load and probe blocks. As these SNRs were different for each participant, they may have resulted in the activation of different perceptual mechanisms for comprehension. Although the labels “hard” and “easy” were chosen to indicate the contrast between the SRT50 and SRT80 to the participants, and although previous research (Koelewijn et al., 2018) and the current study show more self-reported effort during the hard condition as compared to the easy condition, it should be noted that the perceptual and cognitive resources used to achieve the task within one condition may not be homogenous (Baskent et al., 2016; Zekveld et al., 2006).

There are several limitations of the present study to be reported. The trial sequence in the speech-in-noise task only included half a second of retention interval (i.e., a break after the presentation of the sentence and before participants repeated what they heard). This short retention interval did not allow us to observe the full-blown pupil dilation response and therefore the PPD was not included in our pupil metrics. Thus, we were unable to observe the influence of previous load sequence and monetary incentives on the PPD. The observation of larger MPD in the hard SNR condition, nevertheless argues in favor of the MPD capturing listening effort, confirming the validity of our pupillometry set-up and analyses. In addition, monetary incentives may have been insufficient to induce motivation in a sample that was middle aged on average. Future studies could use other forms of incentives (e.g., intrinsic pleasure), which may be more effective in manipulating motivation. Importantly, the current study lacked a control group (or control condition), limiting our conclusions regarding fatigue effects. Future studies can use an additional experimental condition where participants are not fatigued (control condition), or one where participants are fatigued to a greater amount (heavier task load condition).

To conclude, in this study the effect of previous mental load in interaction with motivation and SNR on listening effort was examined. Although easier SNR predicted smaller MPD, monetary incentive and previous load sequence effects on the MPD were not present. Instead, monetary incentives interacted with SNR in influencing BPD, and previous load sequence was shown to decrease the BPD. These results suggest that motivation and previous load may influence the capacity allocated (arousal) in anticipation of listening.
